# Animal Models for Postoperative Implant‐Related Spinal Infection

**DOI:** 10.1111/os.13238

**Published:** 2022-04-25

**Authors:** Yongjie Wang, Mingxue Che, Zhi Zheng, Jun Liu, Xue Ji, Yang Sun, Jingguo Xin, Weiquan Gong, Shibo Na, Yuanzhe Jin, Shuo Wang, Shaokun Zhang

**Affiliations:** ^1^ Department of Spinal Surgery the First Hospital of Jilin University Changchun China; ^2^ Jilin Engineering Research Center for Spine and Spinal Cord Injury Changchun China; ^3^ Institute of Military Veterinary Science the Academy of Military Medical Science of PLA Changchun China; ^4^ Department of Ophthalmology the Second Hospital of Jilin University Changchun China

**Keywords:** Animal models, Implant, Spinal infection, Spine

## Abstract

Postoperative infections following implant‐related spinal surgery are severe and disastrous complications for both orthopaedic surgeons and patients worldwide. They can cause neurological damage, disability, and death. To better understand the mechanism of these destructive complications and intervene in the process, further research is needed. Therefore, there is an urgent need for efficient, accurate, and easily available animal models to study the pathogenesis of spinal infections and develop new and effective anti‐bacterial methods. In this paper, we provide a general review of the commonly used animal models of postoperative implant‐related spinal infections, describe their advantages and disadvantages, and highlight the significance of correctly choosing the model according to the infection aspect under investigation. These models are valuable tools contributing to the better understanding of postoperative spinal infections and will continue to facilitate the invention of novel preventative and treatment strategies for patients with postoperative spinal infections. However, although they are valid and reproducible in some respects, the current animal models present certain limitations. Future ideal spinal infection animal models may assess the bacterial load of the same animal in real‐time *in vivo*, and better mimic the human anatomy as well as surgical techniques. Strains other than *Staphylococcus aureus* account for a large proportion of postoperative spinal infections, and thus, the establishment of models to evaluate other types of microbial infections is expected in the future. Furthermore, novel transgenic models established on advancements in genome editing are also likely to be developed in the future.

## Graphical abstract

In this paper, we provide a general review of the commonly used animal models of postoperative implant‐related spinal infections, describe their advantages and disadvantages, and highlight the significance of correctly choosing the model according to the infection aspect under investigation. These models provide valuable *in vivo* tools to study the pathogenesis of spinal infections and to evaluate the efficacy of a variety of diagnostic tools and treatments.

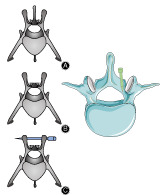

## Introduction

Instrumentation has become an indispensable part of the management of various spinal lesions. Spinal instruments also play an important role in the occurrence of postoperative infections. Postoperative spinal infections are increasing due to more aggressive and frequent spinal surgery^1^, ^2^. Postoperative infection is an important complication of spinal surgery. However, the best imaging method for the diagnosis of postoperative spinal infections has not yet been established. Clinical suspicion of postoperative spinal infections is very important in making a correct diagnosis in the early stage of the disease. To date, during surgical interventions for spinal infections, best practices have not been articulated to optimize health outcomes and resource utilization. Spinal infections are a serious problem for spine surgeons, and there is much debate on how to best use antibiotics and the devices developed so far to treat them. Despite efforts to reduce the infection rate associated with spinal surgery, complications are still common and have greatly increased due to patient comorbidities. Postoperative spinal infections following implant‐related surgery are serious and life‐threatening complications for both orthopaedic surgeons and patients. They can contribute to chronic pain, neurological damage, spinal deformity, disability, and death. The most commonly involved region is the lumbar spine, followed by the thoracic and cervical spine regions. It has also been well‐documented that males are more susceptible than females^3^. This complication is disastrous for healthcare systems because patients need prolonged antibiotic therapies and multiple surgeries as well as long‐term hospitalizations^4^. Managing such complications is extremely expensive, with treatments costing more than $900,000^5^.

Biomechanical protection of the spinal column is a significant issue during and after infections. Different treatment protocols such as antibiotic treatment, debridement, soft tissue care, and implant removal have been developed for postoperative spinal infections with mixed outcomes. Although long‐term and special antibiotic treatments are still the main treatments for spinal implant‐related infections, surgery can provide samples for microbiological identification and histopathological studies, and can also eliminate infections and stabilize the spine. Special attention to the implant and its microbiological culture result will help control postoperative spinal infections. Also, by directly addressing the main causes of pain, surgery provides an opportunity for patients to achieve early pain relief. Over the last decade, spinal surgery has been modified to become minimally invasive, and minimally invasive surgery has been confirmed to have a lower morbidity rate and faster recovery. However, despite the considerable progress in sterile surgery techniques, postoperative care, and the application of antibiotics during the perioperative period, postoperative infections still occur at a fairly high rate, which was reported to be approximately 3%–8% when metal implants were used^1^, ^3^, ^4^, ^6^, ^7^, ^8^, ^9^. This rate is higher among certain patients with operative risk factors. Advanced age, obesity, alcohol abuse, smoking, diabetes, history of cancer, rheumatoid arthritis, hypothyroidism, immunocompromised situations, trauma, and certain pediatric disorders all led to increased infections following spinal surgery^9^, ^10^, ^11^, ^12^. Various factors that may affect the sensitivity to infections have been reported, including the biocompatibility and surface properties of the implant materials^13^, ^14^, ^15^, the stability of the spinal fixation technique^16^, ^17^, ^18^, and the immune status of the patients, as well as the type of bacteria. In revision or multilevel surgeries, intraoperative blood loss, the use of instrumentation, and the rate of postoperative infections can be even higher^6^, ^19^, ^20^, ^21^. The appearance of vancomycin and methicillin‐resistant pathogenic strains adds significant magnitude to surgical infection problems^22^, ^23^, ^24^.

Previous studies showed that bacterial adherence and its ability to induce infections varied with the species and number of bacteria^25^, ^26^. Several previous studies reported that spinal infections were mostly caused by *Staphylococcus aureus* (*S. aureus*)^27^, ^28^, ^29^, ^30^, ^31^, ^32^, ^33^, followed by *Staphylococcus epidermidis*, and *Propionibacterium acnes* ^34^. These bacteria could readily adhere to the foreign implant because orthopaedic implants offer an adhesive substratum surface suitable for the development of biofilm^35^, ^36^. Once bacteria adhere to the implant, they produce a polysaccharide biofilm layer over several days, rendering them insusceptible to antibiotics that are effective in *in vitro* susceptibility tests and host defense mechanisms. Over time, they lead to suppurative inflammation in bone tissue, causing necrosis and resorption of the bone matrix^37^, ^38^, ^39^.

Unlike other orthopaedic fields, there exists no standardized, accepted protocol for the management of spinal infections associated with instrumentation. Spinal infections are challenging because the removal of the infected implant in an instrumented spine often leads to spinal instability and can result in serious neurologic sequelae^20^. Thus, the treatment of postoperative spinal infections remains a challenging problem. To better understand the mechanism of this destructive complication and intervene in the process, further research is needed. Therefore, there is an urgent need for efficient, accurate, and easily available animal models to study the pathogenesis of spinal infections and develop new and effective anti‐bacterial methods. To this end, this paper provides a general review of the various animal models used in the study of postoperative spinal infections and summarizes their weaknesses, advantages, and potential modifications.

## Rabbit Models

In 1998, the first animal model of postoperative spinal infection was established by Guiboux *et al*.^40^. This model was based on a rabbit spine fusion model described by Boden *et al*.^41^, a spinal instrumentation model by Feighan *et al*.^42^, and an intervertebral disk infection model by Guiboux *et al*.^43^. In their study, 20 rabbits were randomly divided into four groups. After anesthesia, an incision was made on the skin and a straight path to L4 and L5 was taken. Then, the fascia was cut longitudinally and the paravertebral muscles were opened to expose the facet joints and lamina. The posterior spinous L4 and L5 processes were removed before laminectomy. Then a figure‐eight 26‐gauge surgical wire was installed bilaterally near the L3/L4 as well as the L4/L5 small joints. After that, all animals received an autogenous bone graft, followed by placing 0.05 mL of 1 × 10^3^ colony‐forming units (CFUs)/mL of *S. aureus* solution onto the transplanted bone and hardware area. The wound was then tightly closed. The rabbits were euthanized 5 days after surgery, and swabs and tissue cultures were taken for infection evaluation. In this study, all rabbits without any preoperative antibiotic treatment were infected, whereas no rabbits that received preoperative or postoperative antibiotics were infected, regardless of hardware implantation.

This model was effective and reproducible. However, there were some problems with the sensitivity of the methods performed, which assessed infection only at one time point using only bacterial cultures. In actual clinical practice, despite the use of prophylactic antibiotics, many patients still have infections, a finding that is not consistent with the results of therapeutic evaluation studies on animals. Insufficient sample sizes and low culture sensitivity may be the reasons for these differences. To improve sensitivity, researchers can extract and culture surgical implants separately from surgical site aerobic swabs and tissue cultures. Further studies could modify this model and evaluate the infection at multiple time points.

**Fig. 1:**
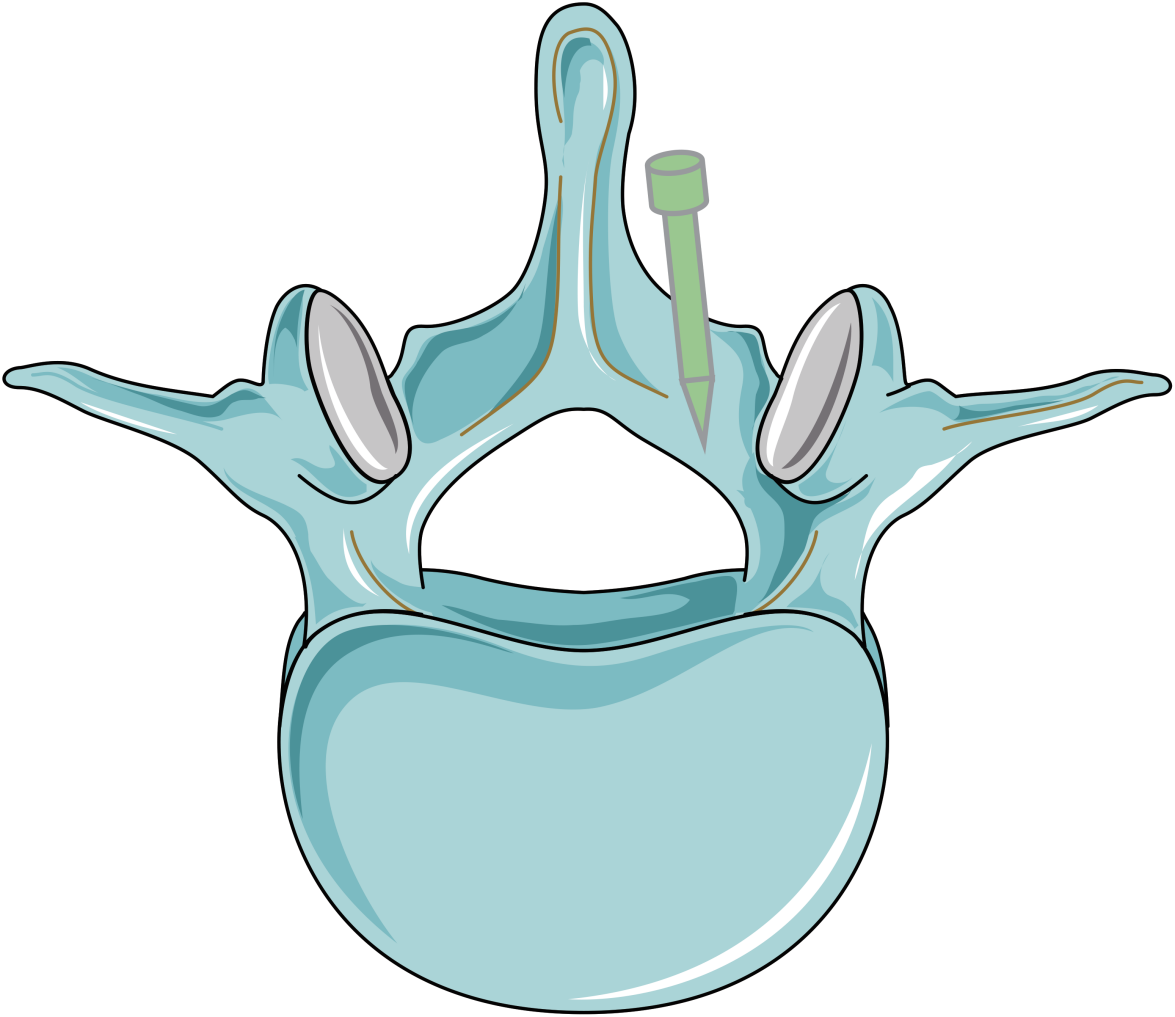
Cranial view on rabbit lumbar vertebra. (A) Normal. (B) After partial laminectomy. (C) After insertion of the stainless steel Kirschner wire.

In 2000, Poelstra *et al*.^44^ invented the novel implant‐related infection rabbit model of methicillin‐resistant *S. aureus* (MRSA). Eight New Zealand white female rabbits (2.5–3.0 kg) were used in this study. After preparation and anesthesia, a 2.5‐cm dorsal incision was cut longitudinally on the skin, then on the fascia until the spinous process was observed. The spinous process was excised to form a hollow defect, simulating partial laminectomy. Then, a 0.85‐mm diameter stainless steel threaded Kirschner wire was inserted into the transverse processes of the L3, L6, and T13 vertebrae (Fig. 1). After that, L6 and T13 were inoculated with 100 μL of sterile saline or MRSA solution in different concentrations (1 × 10^2^, 1 × 10^3^, 1 × 10^4^, or 1 × 10^5^ CFUs) with the L_3_ level serving as the sterile control. Finally, the skin and fascia were tightly closed in layers. On postoperative day (POD) 7, all animals were euthanized, biopsies were utilized to measure spinal infections, and blood and liver were obtained to monitor systemic infections. According to the biopsy cultures, all the sites challenged with 1 × 10^3^ CFUs developed infections, whereas 1 × 10^2^ CFUs did not lead to consistent infections. Therefore, an inoculum of at least 1 × 10^3^ CFUs was thought to establish this infection model successfully. The liver and blood samples exhibited no evidence of systemic sepsis by POD 7. Moreover, none of the control sites developed infections.

This model was established on the separate implantation of three materials in the isolated defect sites of a single animal, simulating posterior spinal instrumentation applied in fusion surgery. In this model, individual sites were inoculated with different CFUs of MRSA to establish a localized and plantation‐centered infection. Soft tissue damage and dead space areas created by partial laminectomy, which are characteristics of posterior spinal implant surgery, were combined in a single model. Dead space areas tend to gather blood, creating a medium for bacteria to replicate and mimicking the human local surgical site environment. This model is unique because several implants and various infectious sites can be assessed simultaneously in the same rabbit. This provides an internal control and makes comparisons between treatment strategies, as well as different implant materials, more convenient. Also, this design decreases the number of animals needed compared to one‐site models. However, more sites of infection will put more pressure on the immune system and may cause potential death. Whether the host's response to infection in this multisite model is different from that of the single‐site model is also controversial.

Several researchers have used this animal model to assess the efficacy of intrawound vancomycin powder in removing bacterial surgical site contamination.^45^ Hazer *et al*.^46^ modified this model to evaluate the antimicrobial effect of polymer‐based silver nanoparticle‐coated pedicle screws. Liu *et al*.^47^ assessed the impact of vancomycin microspheres on reducing *S. aureus* infections. Moreover, Miller *et al*.^48^ evaluated the effect and safety of an autograft and rhBMP‐2 in inducing fusion in postoperative infections based on this model.

In 2017, Laratta *et al*.^49^ established a gram‐negative spinal implant‐related infection model in rabbits. Five female rabbits were used in his study. After the whole back was shaved and anesthesia was conducted, the fourth thoracic (T4), ninth thoracic (T9), first lumbar (L1), and sixth lumbar (L6) levels were marked. Then, the back was cleaned, and lidocaine was injected into all sites for pain control. A 1.5‐cm skin incision was made centered on these levels longitudinally, and then a single incision was undertaken in the fascia. Small retractors were applied to expose the spinous processes, followed by the removal of the dorsal half of the spinous processes, surrounding tissue, and the dorsal aspect of the mammillary body with a rongeur to create a spinal defect. After that, a titanium wire (0.7 mm in diameter and 5 mm in length) was inserted into the designed space to simulate posterior instrumentation. This wire was installed longitudinally in the posterior part between the medial part of the spinous process and the lateral part of the papillary body. Then, a 23‐G needle was utilized to inoculate the implant and wound with 100 μL of bacteria (1 × 10^2^, 1 × 10^3^, 1 × 10^4^, 1 × 10^5^, and 1 × 10^6^ CFUs) or sterile solution. The fascia, sub‐dermis, and skin were tightly sutured in order. Then, the animal was re‐prepared again in a sterile fashion. The same procedure was repeated for the remaining sites on the same animal. All rabbits were euthanized 4 days postoperatively, and the bacterial burden in the implants and surrounding tissue was measured. Blood collection was conducted prior to euthanasia to assess systemic infection. This study showed no evidence of infection in the control sites. An inoculum of 1 × 10^2^ CFUs of *Escherichia coli* (*E. coli*) did not cause a consistent infection, whereas inoculation with 1 × 10^3^ CFUs created a consistent soft tissue infection, but inconsistent infections on the implants. Inoculation with 1 × 10^5^ CFUs of *E. coli* was required to consistently produce both implant and soft tissue infections.

This model is similar to the model of Poelstra *et al*.^44^ with a modification of the instrumentation technique and MRSA replaced with *E. coli*. A limitation was that there was the potential for cross‐contamination between the four sites. Another limitation was that measuring the bacterial burden after 4 days may be too short. Monitoring the bacterial burden for a more extended period would help to determine whether lower doses of bacteria could develop infection over a long time and how the infection changes over time. This animal model could reliably reproduce gram‐negative infections and be utilized to explore the methods for preventing surgical site infections caused by gram‐negative species and guide the future advancement of anti‐bacterial strategies. Some researchers have used this model to assess the efficacy of intrawound tobramycin powder in eradicating bacterial contamination.^50^

In 2020, Gordon *et al*.^51^ established a new rabbit model of *S. aureus* implant‐related spinal infection. Fourteen male Dutch belted rabbits (10–16 weeks) were used in this study. The rabbits were anesthetized, and analgesia was applied prior to surgery. The back skin was shaved and disinfected. Then, a 3‐cm incision was made in the midline longitudinally at the L5 and L6 levels. Subsequently, the fascia was opened to expose the L6 vertebra. The spinous processes and surrounding tissues were entirely removed with a rongeur to produce a hollow defect. Next, orthopaedic‐grade pedicle screws (4 mm long × 1.5 mm wide) were applied to fasten the plate (0.6 mm wide) between two transverse processes. *S. aureus* at different concentrations (1 × 10^2^, 1 × 10^3^, 1 × 10^4^, or 1 × 10^5^ CFUs) was subsequently inoculated onto the surface of the plate and screws. Finally, the surgical sites were sutured. Computed tomography (CT) imaging was conducted to confirm the location of the plate and screws. Before euthanasia, the severity of the spinal infection was evaluated by *in vivo* bioluminescence imaging (BLI), *ex vivo* CFU enumeration, *ex vivo* CT imaging, scanning electron microscopy (SEM) analysis, and ^18^F‐fluorodeoxyglucose positron emission tomography (^18^F‐FDG‐PET). The results showed that the bacterial load in the *ex vivo* bacterial cultures and *in vivo* BLI peaked on day 14. Within 56 days after infection, the biofilm structure could be observed under SEM. ^18^F‐FDG‐PET and CT were used to monitor infection‐mediated inflammation and bone remodeling. PET signals were observed around the implants. CT showed a marked reduction in dense bone and bone remodeling.

This rabbit model can be used as a valuable *in vivo* preclinical research method to study the pathogenesis and new diagnosis and treatment methods before large animal and human studies. Most previous animal models of implant‐associated spinal infection were evaluated in the acute phase (5–14 days) after surgery and infection, and there was no opportunity to assess persistent infections and inflammation in human implant‐associated spinal infections. In this study, advanced *in vivo* BLI and PET/CT imaging techniques were used to measure the bacterial load, infectious inflammation, and bone remodeling at both acute and chronic time points. Orthopaedic‐grade hardware was used in this model, which more closely simulates the spinal implant surgical technique in humans. This study also had several limitations. Longitudinal imaging with ^18^F‐FDG could monitor infectious inflammation and locate the site of infection. However, ^18^F‐FDG is non‐specific and could not distinguish infection‐induced inflammation from non‐infectious inflammation because since ^18^F‐FDG is a marker of glucose uptake, other tissues with high metabolism could be detected. Therefore, in the future, novel PET imaging tracers targeting pathogens or inflammatory response components may help to obtain comprehensive information on the progress of infection^52^. Furthermore, in this study, the assessment of biofilm formation was carried out by SEM, which is limited because it cannot identify the components of the bacteria or extracellular matrix and may actually reflect adherent bacteria. Finally, bone remodeling and bone mineral density changes may be the result of surgery or implantation, rather than a simple infection alone.

## Mouse Models

In 2017, Dworsky *et al*.^53^ established a noninvasive model of implant‐associated infections in mice. In their study, 12‐week‐old C57L/6 wild‐type mice were anesthetized, and a 2‐cm incision was made on the skin and fascia to visualize the right side of the spinous processes. Then, a space for the implant was developed, and a 25‐G needle was implanted through the L4 spinous process. The L‐shaped stainless steel implant (0.1 mm in diameter) was then placed into the defect. The long arm of the implant was longitudinally placed along the spine heading cranially with the short arm in the spine, as shown in our previous study^54^. An inoculation of sterile saline or 1 × 10^2^, 1 × 10^3^, or 1 × 10^4^ CFUs of bioluminescent Xen36 *S. aureus* was applied to the 90° bend of the stainless steel. Then, the wound was carefully closed. After surgery, high‐resolution X‐rays were used to confirm the placement of the implant on POD 0. An *in vivo* bioluminescence imaging system was used to quantify *S. aureus* infections on POD 0, 1, 3, 5, 7, 10, 14, 18, 21, 25, and 35. CFUs were counted on the 35th day after surgery, and bacteria attached to the stainless steel implants and surrounding tissues were quantitatively analyzed.

When the optimal bacterial concentration was determined, 2‐week‐old transgenic mice expressing enhanced green fluorescent protein (GFP) in their myeloid cells (Lys‐EGFP)^55^, ^56^ were inoculated with 1 × 10^3^ CFUs of bacteria or sterile saline. Transgenic mice are considered to have great advantages over wild‐type mice. With the application of Lys‐EGFP mice and the *in vivo* bioluminescence imaging system, researchers could not only measure bacterial loads in real‐time but could also simultaneously assess the immune response. Their study demonstrated that 1 × 10^3^ CFUs were an efficient and safe inoculum to establish infections without local wound breakdown. Furthermore, neutrophil fluorescence peaked on POD 3 before declining in both groups. In the infected group, the immune response was maintained for 35 PODs, indicating that the infection had been established and the neutrophil‐driven inflammatory response was enhanced for 35 days.

This model is powerful and flexible, allowing real‐time study and providing great opportunity for modifications. Another advantage of this model is that it is cheap and efficient, providing multiple data points per animal and avoiding euthanasia of the animals. Most previous animal models required a large number of animals and euthanasia of the animals^40^, ^57^. In recent years, noninvasive *in vivo* imaging has been invented to replace euthanasia‐based models used in the study of infections^58^, ^59^. This technology quantifies and monitors the bacterial load in real‐time without killing the animals. This ability to monitor infection over time has contributed to a greater understanding of various aspects of implant‐related infections^58^, ^60^, ^61^.

However, there are also some limitations to this model. First, this model simplified the surgical steps. All implants used were stainless steel and the implant was installed unilaterally along the spine, affecting the posterior part of the spine only. Installing implants bilaterally or in the anterior part of the spine may affect the interaction between host and bacteria. Different materials used in surgery may show different biocompatibility and susceptibility to bacterial infections. The different materials may also have different effects on the proliferation of different pathogenic microorganisms. At present, most spinal internal fixation materials are titanium alloy or cobalt‐chromium molybdenum alloy. Therefore, animal models using titanium alloy implants are more relevant with the current study of implant‐related infections. Another potential weakness is that the study only applied LysEGFP mice to evaluate immune responses, which only reflects one part of the host immune response as myeloid cells only exist in early immune responses. Therefore, other transgenic mice, such as MacGreen mice, should be included to evaluate the monocyte/macrophage lineages that appear later^62^. Future studies based on this model could thoroughly investigate the host immune response with various types of genetically engineered mice.

Despite the above weaknesses, this study developed a novel spinal implant‐related infection model in mice. It is a powerful tool to study spinal implant infections further. Park *et al*.^63^ used this model to test the efficacy and dose effect of vancomycin powder over an extended time course. This model was also used to evaluate the efficacy of combined antibiotic therapy (vancomycin and rifampin) on postoperative spinal infections^64^. Furthermore, this model was applied to evaluate multimodal imaging for surgical management^65^ and antimicrobial implant coating^66^.

## Rat Models

**Fig. 2:**
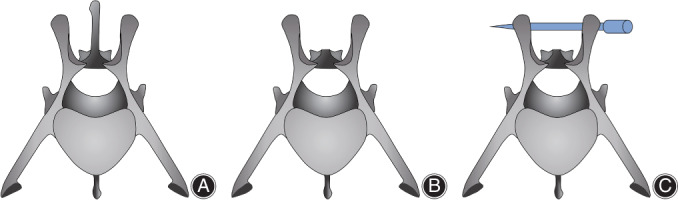
Position of the microscrew in the rat vertebrae.

In 2007, based on the previously designed vertebral fusion model^67^, Ofluoglu *et al*.^57^ successfully set up a pedicle screw *S. aureus* infection model. Forty male Sprague–Dawley rats (300–350 g) were included in this research. A midline incision was conducted in the thoracolumbar area (T10–L1) longitudinally after anesthesia. Then, the paravertebral process and spinous process were separated to expose the facet joint and lamina. The lamina was removed, and a 20‐G needle was used to create a screw entry hole through the junction of the facet joint and lamina. Then, a titanium screw (1 mm wide and 3 mm long) was placed into the pedicle (Fig. 2). A 10‐μL solution of 1 × 10^2^, 1 × 10^3^, or 1 × 10^6^ CFUs of *S. aureus* or sterile saline was applied to the screw head and surrounding tissue. The surgical sites were then closed tightly in layers. All rats were euthanized after 14 days, and later, cultures from the blood, fascia, muscle, and bone were acquired. The bacterial burden was evaluated, and screws were put into 0.5 mL of tryptic soy broth and vortexed, then plated on trypticase soy agar to monitor bacterial growth. Histological examination was also performed on two animals in each group. Based on the histology results, all rats in the *S. aureus* group developed postoperative osteomyelitis. No rats in any group developed a systemic infection. Furthermore, only the 1 × 10^6^ CFU group developed evidence of acute osteomyelitis. These findings demonstrated that 1 × 10^6^ CFUs were the optimal inoculum of *S. aureus*.

The amount of inoculated bacteria ought to be at an appropriate concentration to develop a local bone infection without causing a systemic infection or mortality. Concentrations of 1 × 10^4^ to 1 × 10^9^ CFUs of bacteria were successfully applied to animal models of long bone osteomyelitis according to previous studies^68^, ^69^. This study demonstrated that inoculating 1 × 10^6^ CFUs/10 μL of *S. aureus* after the implantation of a titanium screw was a reproducible model for postoperative spinal infection.

Compared to other types of animals, the advantages of rats are their easy access, maintenance, repeatable results, and robust immune system. Therefore, rats are the most commonly used experimental animal model, especially in long bone and limb infections^70^, ^71^. However, because of their small size and difficulty in surgical manipulations, rats have not been given priority in the spine model. In this study, an operation microscope was applied to facilitate the surgery, and surgical manipulations were easily performed.

In 2013, Cashman *et al*.^72^ modified the rat surgical model of infection using a titanium ligating clip and used this model to assess the ability and efficiency of a new generation of fibrin tissue sealant to deliver antibiotics to the surgical site. This model was also used to determine whether royal jelly had a preventive role in spinal infections in rats^73^.

In 2020, Karau *et al*.^74^ established a biofilm spinal instrumentation rat model with methicillin‐resistant *Staphylococcus epidermidis* (MRSE) to examine the capacity of a local application of vancomycin to treat infection, either suspended within poly (lactic‐co‐glycolic acid) microspheres (MS) or in powder form. The rats were divided into four groups: blank MS, vancomycin MS, vancomycin powder, and no treatment (control), and animal spinal fusion operations were undertaken within 10 days with MRSE biofilms established on implants. In the surgery, the back of every rat was cleaned before an incision was made at the L4–L5 level. Then, the fascia and surrounding tissue were cut along the spinous processes. After the soft tissues were removed, a 24‐G needle was used to produce two similar bone tunnels at the root of the spinous L4 and L5 processes. Then, a flexible stainless steel wire (0.36 mm) was inserted across each tunnel. K‐wires seeded with MRSE were placed on the right side of the spine, and the flexible wires were used to fasten them. The specified treatments were given immediately after the placement of the implant. The animals were euthanized after 8 weeks, and the K‐wires, wire fasteners, bone tissues, and surrounding soft tissues were taken for bacterial cultures.

## Dog Models

In 2009, Chen *et al*.^75^ developed a pyogenic spondylodiscitis model in dogs. Fourteen male dogs were included in the study, weighing 12–15 kg each. After anesthesia, the L1 or L6 positions were marked. The skin was shaved and cleaned, and the desired vertebrae (L1) and discs (T12–L1) were exposed by an anterior retroperitoneal approach. Then, a partial diskectomy was carried out. The end plates of two adjacent vertebrae were removed to make a place for the bacterial inoculum. After that, 4 mL of 5% sodium morrhuate was carefully injected into the bone cavity and surrounding tissues adjacent to the vertebrae. After 1 minute, 100 μL of bacterial inoculum (1 × 10^1^, 1 × 10^2^, 1 × 10^3^, 1 × 10^4^, or 1 × 10^5^ CFUs) or sodium morrhuate was injected into a 1‐cm^3^ gelatin sponge. The sponge was then placed in the previously created intervertebral space. Bone wax was used to seal the remaining defect space to avoid leakage of the inoculum. The fascia and skin were then closed tightly in order. After that, the skin was re‐prepared using iodine, and the procedures of exposure and inoculation were carried out again. All dogs were euthanized 14 days post‐implantation, and the bacterial burden of the tissues was measured. This study suggested that 1 × 10^2^ CFUs was the optimal inoculum concentration. Of the sites infected with 1 × 10^2^ CFUs of *S. aureus*, 90% developed spondylodiscitis of the lumbar spine. Liver samples and blood cultures show no evidence of systematic infections on POD 14. Within 3 days post‐operation, 50% of the animals died after the implantation of suspensions at concentrations higher than 1 × 10^3^ CFUs.

The more sites in an animal that are compared, the fewer animals are needed for the study. Therefore, one advantage of this dog model is that it is based on inoculating bacteria into separate lumbar intervertebral spaces in a single dog. Different sites were administrated different concentrations of bacteria to produce consistent pyogenic spondylodiscitis. Another advantage is that the inoculum concentration used was lower than that previously reported. Surgical trauma, bone wax or gelatin sponge, the dead space deliberately created, and a unique local blood supply may contribute to the differences in the concentrations. Additionally, compared to rodents, dogs are more comparable to humans in body shape, anatomy, physiology, metabolism, immunology, and genetics. Sequencing of the canine genome (99% complete, ∼2.5 billion base pairs)^76^ has shown that there are greater similarities between dog and human gene sequences than between humans and mice^77^. Therefore, among all the listed animal models, this canine osteomyelitis model is most similar to human beings in terms of the immune system and anatomy of the spine. It is similar to human spinal diseases in many aspects and can be used as a vehicle for the study of prevention and treatment methods. Chen *et al*.^78^ used this model to confirm the presence, type, and origin of bacteria adhering metal implants in the infected region. Furthermore, this model was used to study differences between *Mycobacterium tuberculosis* and *Staphylococcus aureus* in their capability to induce implant‐related infections^79^.

There are also some limitations to this model. First, although sodium morrhuate has been used extensively to cause local infections because of its surfactant properties and high arachidonate content^69^, ^80^, ^81^, ^82^, ^83^, previous studies showed that sodium morrhuate affected the replication of *S. aureus* ^69^. Therefore, in future research, strategies should be taken to eliminate its toxic effect on *S. aureus*. Second, it may not precisely represent the real clinical features of pyogenic spondylodiscitis because patients with pyogenic spondylitis often have a low immune function or severe medical comorbidities. Therefore, it is difficult to mimic the complicated clinical scenarios of spinal infections.

## Conclusions and Future Directions

The contribution of animal models to the study of human spinal infections has been a fundamental and critical part of the development of effective therapies. This article provides a general review of the different characteristics of animal models commonly used to study postoperative spinal infections, and each model presents advantages and/or disadvantages (Table 1). These models were all established to study the pathogenesis, diagnosis of infections in the spine, and test the efficacy of various interventions. Apart from being reproducible, these animal models simulate many aspects of postoperative spinal infections and help to explain the mechanisms of this disease, among which the dog model is most similar to human spinal diseases. Despite some specific limitations, these models are valuable tools contributing to the better understanding of postoperative spinal infections. These models will continue to facilitate the invention of novel preventative and treatment strategies for patients with postoperative spinal infections. In the future, ideal spine infection animal models may better mimic human anatomy as well as surgical techniques, which can also assess the bacterial burden of the same animal at different time points through *in vivo* imaging technology. Additionally, most current animal models use *S. aureus* or MRSA as the pathogen to evaluate postoperative infections. Therefore, future models ought to use other bacterial species as other bacteria account for a significant proportion of postoperative spinal infections. Moreover, novel transgenic models established on advancements in genome editing are likely to be developed in the future.

**Table 1:** Summary of postoperative implant‐related spinal infection animal models

Animal models	Bacteria species	Surgical procedure	Evaluation technique	Advantages	Disadvantages
Rabbit (Guiboux *et al*., 1998)^40^	*S. aureus*	A figure‐eight 26‐gage wire was installed bilaterally around the L_3_/L_4_ and L_4_/L_5_ small joints, followed by placing 0.05 mL of 1 × 10^3^ CFUs *S. aureus*/mL solution onto the bone transplanted and hardware area.	*Ex vivo* CFUs enumeration	•Effective and reproducible •Mimicking the clinical surgical technique accurately •Large size and docility allow for easy operation and sampling	•Not consistent with real clinical practice •Small sample size •Low sensitivity of method •No long‐term monitoring •No internal control
Rabbit (Poelstra *et al*., 2000)^44^	MRSA	Insertion of a stainless steel threaded Kirschner wire into the transverse processes of L_3_, L6, and T13 vertebrae. After that, T13 and L6 were inoculated with 100 μL sterile saline or MRSA in different concentrations (1 × 10^2^, 1 × 10^3^, 1 × 10^4^, or 1 × 10^5^ CFUs). The L3 level was used as control.	*Ex vivo* CFUs enumeration	•Reproducible and effective •Mimicking clinical surgical technique accurately •Large size for easy operation •Having an internal control •Multiple implants and sites can be evaluated simultaneously •Decreasing the animal number	•No long‐term monitoring •Host response to infection may be different from one‐site model •Multiple sites of infection put more pressure on the immune system •Possibility of cross contamination
Rabbit (Laratta *et al*., 2017)^49^	*E. coli*	A titanium wire (0.7 mm in diameter and 5 mm in length) was inserted into the designed spinal defect. The wire was implanted longitudinally in the posterior part between the medial part of the spinous process and the lateral part of the papillary body. Then a 23 G needle was used to inoculate the implant and wound with 100 μL bacteria.	*Ex vivo* CFUs enumeration and visual assessment	•Effective and reproducible •Mimicking the human local surgical site environment •Large size and docility allow for easy operation and sampling •Having an internal control •Multiple sites can be evaluated simultaneously in the same rabbit •Decreasing the animal number	•Possibility of cross contamination •Internal control may not be representative of non‐infectious area •Host response to infection may be different from one‐site model •Multiple sites put more pressure on the immune system •No long‐term monitoring
Rabbit (Gordon *et al*., 2020)^51^	*S. aureus*	Orthopaedic‐grade pedicle screws (4 mm length ×1.5 mm width) were applied to fix the plate (0.6 mm width) between two transverse processes. Then, *S. aureus* in different concentrations were subsequently inoculated on the surface of the plate and screws.	*In vivo BLI*, SEM, *ex vivo* CFUs enumeration, *ex vivo* CT imaging and ^18^F‐FDG‐PET	•Effective and reproducible •Mimicking clinical surgical technique accurately •Large size for easy operation •Multiple sites can be evaluated simultaneously •Decreasing the animal number •*In vivo* bacterial load, inflammation and bone remodeling could be assessed in both acute and chronic time points	•Multiple sites of infection put more pressure on the immune system •Could not distinguish infection‐induced inflammation from non‐infectious inflammation •Bone remodeling and changes in bone density may be a result of surgery or implants rather than infection alone
Mouse (Dworsky *et al*., 2017)^53^	*S. aureus*	A 25 G needle was inserted through the L_4_ spinous process. A L‐shaped 0.1 mm diameter stainless steel implant was put into the defect. Then, bioluminescent Xen36 *S. aureus* was applied onto the 90° bend of the implant.	*In vivo BLI*, *ex vivo* CFUs enumeration	•Flexible, cheap, and efficient •Study of host response and bacteria in real time •Long‐term monitoring •Providing multiple data point per animal	•The implant was placed unilaterally involving only the posterior elements of the spine •Only reflect one part of the host immune response •Difficulty in surgical manipulations
Rat (Ofluoglu *et al*., 2007)^57^	*S. aureus*	A 20 G needle was used to create a screw entry hole through the junction of the facet joint and lamina. Then a titanium screw (1 mm diameter and 3 mm length) was placed into the pedicle. A 10 μL solution of *S. aureus* was applied on screw head and surrounding tissues.	Histological examination, *ex vivo* CFUs enumeration	•Reproducible and cheap •Surgical technique greatly mimics implantation of pedicle screws	•No long‐term monitoring •No internal control •Requiring large number of animals •Biofilm formation was not evaluated •Difficulty in surgical manipulations
Rat (Karau *et al*., 2020)^74^	MRSE	A 24 G needle was used to produce two bone tunnels at the spinous processes on L_4_‐L_5_. Then a flexible stainless steel wire was inserted into each tunnel. K‐wires seeded with MRSE were placed on the right side of the spine.	*ex vivo* CFUs enumeration, SEM	•Reproducible and effective •Accurately mimicking human spinal infection	•Difficulty in surgical manipulations •No internal control •Requiring many animals •No long‐term monitoring
Dog (Chen *et al*., 2009)^75^	*S. aureus*	Partial diskectomy was carried out to make a place for the bacterial inoculum. Then, 4 mL of 5% sodium morrhuate was injected into the bone cavity and surrounding tissues. After 1 minute, 100 μL bacterial inoculum or Sodium morrhuate was injected into a 1‐cm^3^ gelatin sponge. The sponge was then placed in the previously created intervertebral space. The bone wax was used to seal the defect space to avoid the inoculum leaking out.	Histological examination, *ex vivo* CFUs enumeration	•Multiple sites can be evaluated simultaneously in the same dog •Having an internal control •Inoculum concentration was lower •Most similar to the human being in the terms of immune system and anatomy of spine •Decreasing the animal number •Easy to manipulate	•Morrhuate solutions influence the duplicate of *S. aureus*. •May not represent the real clinical features of pyogenic spondylodiscitis •No long‐term monitoring •Possibility of cross contamination •Multiple sites of infection put more pressure on the immune system

*S. aureus, Staphylococcus aureus*; CFUs, colony‐forming units; MRSA, methicillin‐resistant *S. aureus*; *E. coli*, *Escherichia coli*; BLI, bioluminescence imaging; SEM, scanning electron microscopy; CT; computed tomography; ^18^F‐FDG‐PET, ^18^F‐fluorodeoxyglucose positron emission tomography; MRSE, methicillin‐resistant *Staphylococcus epidermidis*.

### Authorship declaration

We acknowledge that all authors listed meet the authorship criteria according to the latest guidelines of the International Committee of Medical Journal Editors and all authors are in agreement with the manuscript.

## Disclosure Statement

The authors have declared that there are no competing interests.
